# Examining the educational impact of the mini-CEX: a randomised controlled study

**DOI:** 10.1186/s12909-021-02670-3

**Published:** 2021-04-21

**Authors:** Susanne Skjervold Smeby Martinsen, Torvald Espeland, Erik Andreas Rye Berg, Eivind Samstad, Børge Lillebo, Tobias S. Slørdahl

**Affiliations:** 1 Department of Clinical and Molecular Medicine, Norwegian University of Science and Technology (NTNU), Trondheim, Norway; 2 Department of Circulation and Medical Imaging, Norwegian University of Science and Technology (NTNU), Trondheim, Norway; 3 Clinic of Cardiology, St. Olavs Hospital, Trondheim University Hospital, Trondheim, Norway; 4 Clinic of Thoracic and Occupational Medicine, St. Olavs Hospital, Trondheim University Hospital, Trondheim, Norway; 5 Clinic of Medicine and Rehabilitation, Ålesund Hospital, Møre og Romsdal Hospital Trust, Ålesund, Norway; 6 Clinic of Medicine and Rehabilitation, Levanger Hospital, Nord-Trøndelag Hospital Trust, Levanger, Norway; 7 Department of Haematology, St. Olavs Hospital, Trondheim University Hospital, Trondheim, Norway

**Keywords:** Medical education research, Formative assessment, Feedback, Workplace-based assessment

## Abstract

**Background:**

The purpose of this study is to evaluate the mini-Clinical Evaluation Exercise (mini-CEX) as a formative assessment tool among undergraduate medical students, in terms of student perceptions, effects on direct observation and feedback, and educational impact.

**Methods:**

Cluster randomised study of 38 fifth-year medical students during a 16-week clinical placement. Hospitals were randomised to provide a minimum of 8 mini-CEXs per student (intervention arm) or continue with ad-hoc feedback (control arm). After finishing their clinical placement, students completed an Objective Structured Clinical Examination (OSCE), a written test and a survey.

**Results:**

All participants in the intervention group completed the pre-planned number of assessments, and 60% found them to be useful during their clinical placement. Overall, there were no statistically significant differences between groups in reported quantity or quality of direct observation and feedback. Observed mean scores were marginally higher on the OSCE and written test in the intervention group, but not statistically significant.

**Conclusions:**

There is considerable potential in assessing medical students during clinical placements and routine practice, but the educational impact of formative assessments remains mostly unknown. This study contributes with a robust study design, and may serve as a basis for future research.

**Supplementary Information:**

The online version contains supplementary material available at 10.1186/s12909-021-02670-3.

## Background

Along with the adoption of competency-based education programmes, there has been increasing emphasis on workplace-based assessments (WBAs) in medical education [1, 2]. WBAs are assessments that assess clinical competence and professional behaviour in everyday practice. As WBAs require direct observation of trainees in the workplace, they also provide opportunities for feedback, and are therefore increasingly being used as methods of formative assessment [3].

The mini-Clinical Evaluation Exercise (mini-CEX) is one of the most commonly used WBAs, and since its introduction in 1995 has been implemented in both undergraduate and postgraduate programmes worldwide [1, 4–7]. Trainees are observed and evaluated while performing a history or physical examination, followed by structured feedback [3, 8]. The mini-CEX can be used with a wide range of clinical problems and workplace settings, allowing trainees to receive feedback from different supervisors [3]. The mini-CEX evaluates multiple competencies that are important in high-quality care [3].

The mini-CEX remains among the most studied WBAs with regards to reliability and validity as an assessment tool [1]. Research has shown that acceptable reliability can be achieved with eight to ten encounters, but the exact number will naturally vary with the stakes and purpose of the assessment [9]. The close correspondence between assessment and practice setting limits validity threats such as construct-irrelevant variance and construct underrepresentation [9]. There are also consistent findings of positive correlations with other assessment outcomes, including high-stakes national specialty examinations [7, 9–12]. Additionally, a number of studies report higher scores with each year of postgraduate training or improvement in scores throughout the academic year [4, 8, 9, 13, 14]. However, concerns have been raised against the scoring component of the mini-CEX [9]. These are primarily rater leniency, high intercorrelations on the individual competencies, and limited research into the effects of rater training.

Evidence is limited for its consequential validity as a formative assessment tool. As the mini-CEX and other WBAs are increasingly being used for providing feedback to trainees in order to support learning and development, research into the impact on educational outcomes would constitute an important source of validity [15]. A systematic review of the educational impact of the mini-CEX found that the majority of articles presented effects on learner perceptions [15]. Only two articles reported on acquisition of knowledge and skills, and demonstrated positive effects on trainee performance in summative clinical examinations [16, 17]. However, as these studies were sequential cohort studies, drawing conclusions concerning causality is difficult.

The aim of this study was to compare mini-CEX assessments with traditional ad-hoc feedback in order to examine its educational impact, effects on direct observation and feedback, as well as student perceptions of the mini-CEX as a formative assessment tool.

## Methods

### Study design

We conducted a cluster randomised controlled trial with two groups and blinded outcome assessment. A cluster trial design was chosen to avoid contamination (doctors who received extra training in assessment and feedback using the mini-CEX could not be expected to treat individual students differently), as well as for practical purposes.

### Study setting

The six-year undergraduate medical programme at the Norwegian University of Science and Technology (NTNU) is integrated and problem-based. Students cover most clinical subjects in Years 3 and 4. The following year, they complete a 16-week clinical placement at one of the general hospitals in the region, during which this study took place in 2018. This undergraduate setting was chosen as it allows for better standardisation of what is learned during these weeks, and made organising post-study assessments easier.

The clinical placement includes general medicine (7 weeks), general and orthopaedic surgery (7 weeks) and anaesthesia (2 weeks), and all students are required to complete the same checklist of activities and procedures. Prior to this study, feedback had not been formalised in WBAs and was given on an ad-hoc basis. That is, immediate feedback given by doctors or other health professionals while working with students, or prompted by students asking for feedback or help.

### Participants and randomisation

**Fig. 1:**
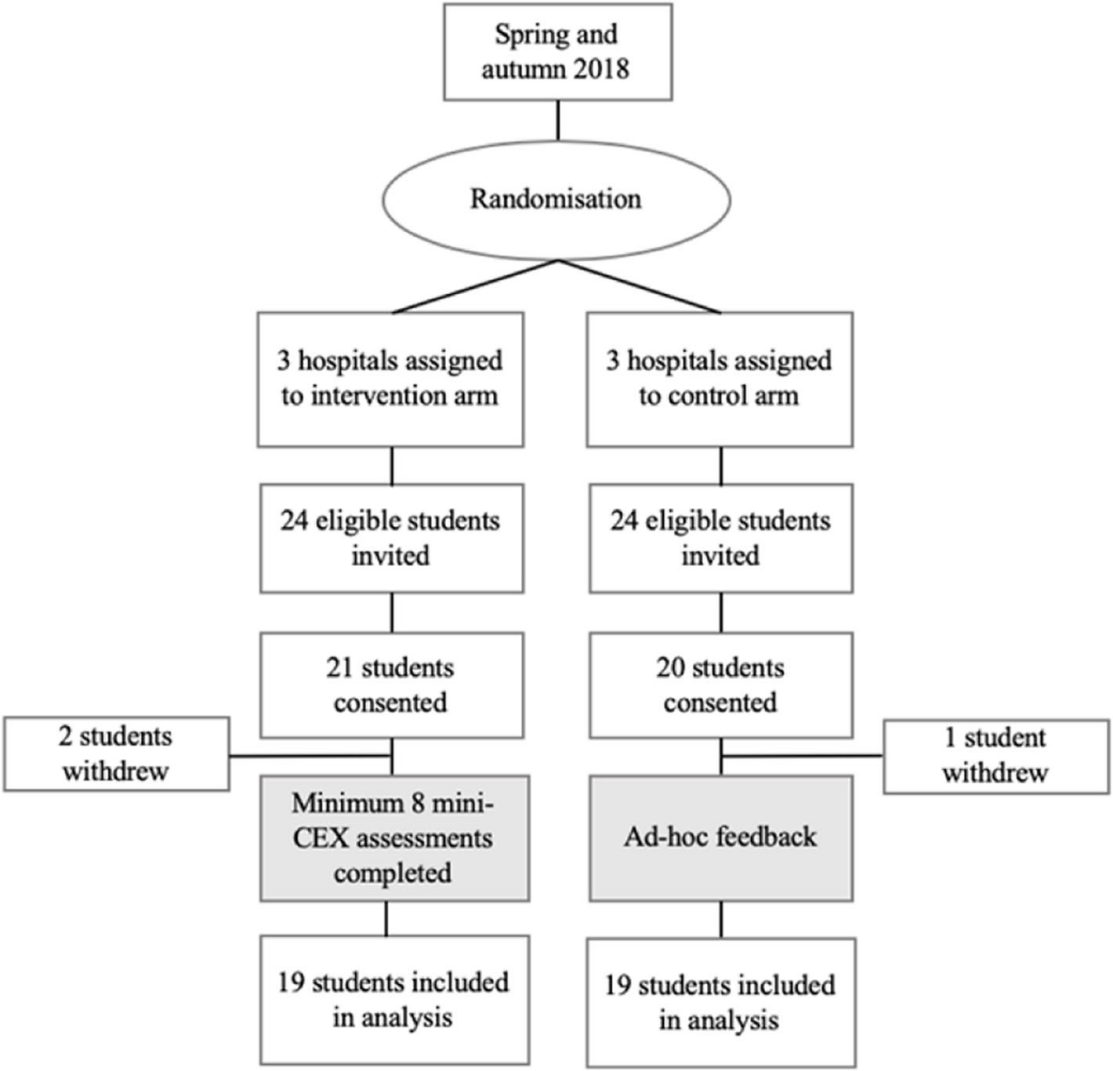
Flow chart of randomised controlled study. Mini-CEX: mini-Clinical Evaluation Exercise

Six of the nine general hospitals in the region were enrolled in the study (Fig. 1). The six hospitals were allocated in a 1:1 ratio to give feedback using mini-CEX assessments (intervention arm) or continue with ad-hoc feedback (control arm), using a simple randomisation procedure by means of drawing lots. Student participation was voluntary and there were no exclusion criteria. All participants provided written consent. The study was approved by the Norwegian Centre for Research Data (project number: 56646).

Forty-eight students were invited by email, and of these, 41 students consented to participate. Three students later withdrew from the trial because they were unable to attend outcome assessments, leaving 19 students in the intervention group and 19 students in the control group that were included in the analyses.

### Intervention

Participants in the intervention group were asked to complete a minimum of eight formative mini-CEX assessments. They were encouraged to choose patients with a wide range of clinical problems and assessors with different levels of training (newly qualified doctors to consultants). Apart from mini-CEX assessments, no other changes were made to their clinical placement. The amount of time spent in clinical practice, and requirements with regards to checklist activities and procedures remained the same between the groups.

The assessment part of the mini-CEX consists of six competencies and one overall score [13]. Each competency is scored on a nine-point rating scale. The feedback part consists of one box for ‘Especially Good’ and one for ‘Suggestions for Improvement’.

All participants and assessors were naïve to the mini-CEX. Thus, a 45-min session was held for doctors in each intervention hospital. It emphasised the importance of direct observation and effective feedback. Using a video recording, doctors completed a mini-CEX assessment, followed by a plenary discussion. A written guide was also provided.

Students in both groups were given a presentation of the study aims and outcome assessments, in addition to written material included in the invitation email. Students in the intervention group were also given the same introduction to the mini-CEX as was held for the doctors in the intervention hospitals.

### Outcome measures

At the end of the clinical placement, all participants completed a survey, a written test and an Objective Structured Clinical Examination (OSCE). These assessment methods were chosen because they are familiar to students from the university’s assessment programme, but were held separately and did not have any consequences for the students’ progression.

The OSCE consisted of six eight-minute stations (Table 3). Station topics were chosen based on common patient presentations to emergency departments (i.e., chest pain, dyspnoea, fever, abdominal pain, limb injury and neurological symptoms). All stations were drafted by the first author, and reviewed and edited by content experts. Standardised patients were trained in each specific clinical scenario, and remained the same throughout the study. The stations were filmed and later checklist-scored by two independent examiners, blinded to the intervention.

The written test consisted of 43 single best answer multiple choice questions (MCQs). Most items were selected from previously used examination items, with item difficulty of 0.20–0.80 and item discrimination index above 0.20. Tests were corrected without negative marking or corrections-for-guessing [18].

The first part of the survey was answered by both groups, and consisted of 40 Likert-type questions and 4 free text questions divided into three sections: (a) perceptions of feedback, (b) perceptions of learning and confidence, and (c) perceptions of motivation. A review of the literature on feedback, especially the work of Hattie and Timperley, informed the design [19]. Items were constructed adhering to best practices for item-writing and item-design [20]. To ensure that questions were unambiguous and meaningful, cognitive interviews utilising the probing method were held with students who had recently completed their clinical placement [21].

The second part of the survey was answered only by the intervention group and comprised of 13 items on perceptions of the mini-CEX, adapted with permission from Bindal and colleagues [22]. There were eight Likert-type questions, four tick box questions and one free text question.

### Statistical analyses

Analyses of student learning and perceptions were based on individual student-level data, rather than on the cluster-level summarised data. Students select their hospital on the basis of a randomly assigned number which ensures some degree of randomisation. Data from previous examinations indicated that a total of 17 students in each arm for the OSCE and 29 students in each arm for the written test, were needed to achieve an 80% power to detect a 5% difference in test scores with a 2-sided 0.05 significance level.

One-way analysis of variance (ANOVA) was used to compare intervention and control group mean scores on the OSCE and written test. Since the trial was cluster randomised, a second analysis was performed using a one-way analysis of covariance (ANCOVA), controlling for previous examination scores to account for baseline differences in students’ clinical skills and knowledge. For the OSCE, mean Z-scores of the three previous summative OSCEs in Years 3 and 4 were used as the covariate. For the written test, mean Z-scores of the three previous summative written examinations in Years 3 and 4 were used as the covariate.

Interrater reliability for the two examiners on the OSCE was calculated using a two-way random absolute agreement intraclass correlation (ICC2,2) [23]. The reliability of the total score was calculated based on the mean of the two examiners’ scores using Cronbach’s alpha. Reliability of the written test was calculated using the Kuder-Richardson Formula 20. Item difficulty was given by the proportion of students who answered individual items correctly, and item discrimination by the Point-Biserial Correlation.

The first part of the survey was divided into one question (seniority of doctors providing feedback) and five scales (quantity of feedback, quality of feedback, learning, confidence, and motivation) consisting of 3–11 items. Three items were removed to improve internal consistency of scales, which were calculated using Cronbach’s alpha. Mann-Whitney U-tests were used to compare groups because of non-normality of data. Correction for multiple comparisons was not performed on the basis that this study is considered preliminary, and all comparisons were planned ahead and reported in their entirety. Statistical analyses were performed using IBM SPSS Statistics 25 (SPSS Inc., Chicago, IL, USA).

Free text answers on the second part of the survey (mini-CEX) were analysed using Systematic Text Condensation (STC) according to Malterud’s description [24]. NVivo 11 (QSR International Pty Ltd., Melbourne, Australia) was used to conduct the analysis.

## Results

### Characteristics of mini-CEX encounters

A total of 160 mini-CEX assessments were collected and analysed (Table 1). Each participant completed a mean number of 8.4 mini-CEX assessments (standard deviation 0.8; range 8–10). Of the 160 encounters, 54% occurred in general medicine, 43% in general surgery and orthopaedics, and 3% in anaesthesiology. For additional characteristics, see Additional file 1.

**Table 1:** Characteristics of mini-CEX assessments

	*Frequency* (% of total)^a^	Mean (SD)	Range
**Assessment**			
History taking	117 (73.1)	7.55 (1.19)	3–9
Physical examination	113 (70.6)	7.40 (1.26)	3–9
Professionalism	158 (98.8)	8.04 (1.00)	5–9
Clinical reasoning	77 (48.1)	7.44 (1.27)	3–9
Counselling	50 (31.3)	7.50 (1.28)	4–9
Organisation/efficiency	128 (80.0)	7.34 (1.36)	3–9
Overall	114 (71.3)	7.71 (0.99)	5–9
**Feedback**			
Especially good	135 (83.8)		
Suggestions for improvement	112 (70.0)		
**Time**			
Observation (minutes)	149 (93.1)	19.8 (14.7)	2.0–90.0
Feedback (minutes)	140 (87.5)	5.6 (4.5)	0–30.0
**Total no. of mini-CEXs**	160 (100.0)		

*Note:* ^a^ denotes the number of mini-CEX forms (and percent of total number of forms) on which each competency, feedback or time spent was recorded.

### Student perceptions of mini-CEX assessments

The majority (79%, 15/19) of participants in the intervention group were positive or very positive to the use of mini-CEX assessments during their clinical placement (Table 2). About 58% (11/19) of participants found mini-CEX assessments useful or very useful in their clinical placement. Only three participants found the assessments useless.

**Table 2:** Responses to survey on mini-CEX assessments

	Mean (SD)
Expectations for the use of mini-CEX^a^	4.2 (0.9)
Confidence that mini-CEX is a true reflection of your abilities^b^	2.9 (1.0)
Ease of finding doctors to conduct mini-CEX^c^	3.2 (0.7)
Usefulness of mini-CEX in clinical placement^d^	3.5 (1.0)
	*N* (% of respondents)
**Planning of mini-CEX**	
Pre-planned	13 (68.4)
Ad hoc/on the job	6 (31.6)
Retrospective	0 (0.0)
**Time taken after mini-CEX to receive feedback**	
Immediately	9 (47.4)
< 30 min	9 (47.4)
< 2 h	1 (5.3)
> 2 h	0 (0.0)
**Time taken after mini-CEX to receive form**	
Immediately	9 (47.4)
< 30 min	10 (52.6)
< 2 h	0 (0.0)
> 2 h	0 (0.0)
**Doctor refuse to carry out mini-CEX**	
Yes	4 (21.1)
No	15 (78.9)

^a^*1* Very negative, *2* negative, *3* neutral, *4* positive, *5* very positive

^b^*1* Very unconfident, *2* unconfident, *3* neutral, *4* confident, *5* very confident

^c^*1* Very difficult, *2* difficult, *3* neutral, *4* easy, *5* very easy

^d^*1* Very useless, *2* useless, *3* neutral, *4* useful, *5* very useful

A minority of the participants reported that a doctor had refused to do an assessment. Reasons were being too busy (100%, 4/4), and lack of training and not being confident in how to perform assessments (25%, 1/4).

Ninety-five percent (18/19) of participants gave free text comments on the use of mini-CEX. Analysis of comments fell within two main themes, each with two subthemes: *Feedback* (usefulness of feedback, forcing observation and feedback) and *feasibility* (difficulty conducting assessments, need for assessor training).

#### Usefulness of feedback

Participants were divided in their perception of the usefulness of mini-CEX assessments. Some commented that feedback had been very valuable for their learning and development, and wished they had done more assessments. Others commented that feedback had been less useful than expected. Many participants commented that they would have liked to receive more constructive feedback on what could be improved: “*I have found [assessments] very useful, especially when assessors have taken the time to give both positive and constructive feedback. Some assessors did not come up with any suggestions for improvement, whereby it loses its purpose.”* Some participants felt that feedback from more experienced doctors, such as specialty registrars and consultants, was or would have been more useful.

#### Forcing observation and feedback

Some participants remarked on the value of mini-CEX assessments in terms of ‘forcing’ observation and feedback: “*Mini-CEX assessments are a fantastic way of ‘forcing’ doctors to observe you conducting a clinical examination or history.”* One participant also commented that assessments made asking for constructive feedback easier, because it was part of the form.

#### Difficulty conducting assessments

Many participants felt that finding a time or suitable clinical setting was challenging, especially as assessors were often too busy. Some participants pointed out that ease of conducting assessments varied between departments, medicine being easier than surgery. Some participants stated they would have liked doctors to suggest performing mini-CEX from time to time.

#### Need for assessor training

Some participants experienced that doctors did not have training in how to conduct assessments and give feedback.

### Impact on clinical skills (OSCE) and knowledge (written test)

Characteristics of the OSCE are presented in Table 3. Mean total score based on the average of the two examiners’ scores was 116.1 (65.2%). Mean percentage scores on stations ranged from 61.5% (Station 1) to 75.3% (Station 3). Interrater reliability was found to be 0.92 and Cronbach’s alpha was 0.69 for total test scores. For the written test, the mean total score was 21.8 (50.8%) and reliability (KR-20) was 0.44. Mean item difficulty was 0.51 and mean item discrimination (point-biserial correlation) was 0.20.

**Table 3:** Characteristics of OSCE

Station	Topic	Skills assessed	Total score possible	Examiner 1 mean raw score (SD)	Examiner 2 mean raw score (SD)	Cronbach’s alpha if item deleted^a^
1	Febrile neutropenia	H, CR	30	18.9 (2.5)	18.0 (2.4)	0.64
2	Ruptured AAA	PE, CR	22	20.4 (2.7)	20.5 (2.9)	0.64
3	Transient ischaemic attack	PE, CR	32	24.0 (3.2)	24.2 (2.6)	0.65
4	Tachycardia-induced myopathy	H, CR	30	14.1 (1.8)	14.1 (1.8)	0.67
5	Pulmonary embolism	H, CR	32	17.6 (2.7)	17.2 (3.2)	0.63
6	Osteoarthritis of the hip	PE, CR	32	20.9 (4.1)	22.4 (3.3)	0.68
						Cronbach’s alpha^a^
Total			178	115.8 (10.9)	116.4 (9.9)	0.69

*AAA* abdominal aortic aneurysm, *H* history taking*, PE* physical examination*, CR* clinical reasoning

^a^Cronbach’s alpha calculation based on the mean of the two examiner scores

Table 4 compares mean percentage scores on the OSCE and written test between the intervention and control group. Observed mean scores on the OSCE were 3.4% higher in the intervention group. When past OSCE Z-scores were controlled for, the difference between the group means decreased to 2.4%. Neither of these were statistically significant.

**Table 4:** Comparison of mean percentage scores on OSCE and written test between intervention and control group

	*N*	Observed mean % score (SD)	ANOVA	Adjusted mean % score (SE)	ANCOVA
**OSCE**					
Intervention	19	0.669 (0.053)	F = 3.603, *p* = 0.066	0.664 (0.012)^a^	F = 1.884, *p* = 0.179^b^
Control	19	0.635 (0.056)		0.640 (0.012)^a^	
**Written test**					
Intervention	19	0.532 (0.090)	F = 2.674, *p* = 0.111	0.525 (0.020)^c^	F = 1.395, *p* = 0.245^d^
Control	19	0.484 (0.094)		0.491 (0.020)^c^	

^a^Adjustments based on mean Z-scores of past OSCE = 0.102; ^b^Homogeneity of regression tested and not significant: F = 0.088, p > 0.05; ^c^Adjustments based on mean Z-scores of past written examinations = 0.029; ^d^Homogeneity of regression tested and not significant: F = 0.552, *p* > 0.05

Observed mean scores on the written test were 4.8% higher in the intervention group. When past written examination Z-scores were controlled for, the difference between the group means decreased to 3.4%. Neither of these were statistically significant.

### Perceptions of direct observation, feedback and learning

Both groups reported that doctors in their first year of training most frequently provided feedback and supervision. More experienced junior doctors and consultants provided feedback to a lesser extent.

Table 5 presents a summary of survey items and scales. There was good internal consistency in the data looking at the entire scale with a Cronbach’s alpha of 0.84. There were no statistically significant differences between the two groups with respect to the five scales. Statistically significant differences were found for only two of the survey items: feedback on history taking was more commonly reported in the intervention group, and students in the intervention group perceived their own ability to identify normal and abnormal findings higher than those in the control group.

**Table 5:** Survey scales with comparisons of mean scores between intervention and control group

Scale	Cronbach’s alpha	Intervention group, mean (SD)	Control group, mean (SD)	Mann-Whitney U test
**Quantity of feedback**	**0.61**	**2.5 (0.4)**	**2.4 (0.5)**	***p*** **= 0.39**
History taking^1^		3.0 (0.6)	2.2 (0.7)	*p* < 0.01*
Physical examination^1^		2.8 (0.6)	2.5 (0.6)	*p* = 0.15
Procedures^1^		3.0 (0.7)	3.0 (0.7)	*p* = 0.84
Clinical reasoning^1^		2.4 (0.7)	2.7 (0.7)	*p* = 0.21
Presenting findings/cases^1^		2.3 (0.7)	1.9 (0.9)	p = 0.21
Satisfaction with amount of feedback^2^		2.5 (0.9)	2.5 (1.0)	*p* = 0.77
Would have liked more feedback^a, 2^		1.4 (0.5)	1.7 (0.9)	*p* = 0.37
**Quality of feedback**^2^	**0.75**	**3.1 (0.6)**	**3.3 (0.6)**	***p*** **= 0.64**
Direct observation		2.3 (0.9)	2.7 (1.0)	*p* = 0.16
Positive feedback		3.7 (0.7)	3.2 (0.9)	*p* = 0.08
Constructive, negative feedback		2.8 (0.9)	2.7 (0.7)	*p* = 0.71
Guidance on how to improve		3.3 (0.9)	3.4 (0.8)	*p* = 0.73
Wide range of patients		3.0 (1.2)	3.0 (1.0)	*p* = 0.86
Quality of feedback		3.0 (0.9)	3.3 (0.9)	*p* = 0.44
Usefulness of feedback		3.6 (1.0)	4.0 (0.9)	*p* = 0.28
Feedback made me learn more		3.5 (0.9)	4.1 (1.0)	*p* = 0.09
**Learning**^2^	**0.64**	**3.9 (0.3)**	**3.8 (0.4)**	***p*** **= 0.58**
Identifying key information in the history		4.1 (0.5)	3.8 (0.8)	*p* = 0.25
Efficiency in history taking		4.2 (0.7)	4.1 (0.8)	*p* = 0.75
Structured clinical examination		4.2 (0.9)	4.0 (0.7)	p = 0.25
Efficiency in clinical examination		4.2 (0.6)	4.1 (0.8)	p = 0.86
Identifying normal and abnormal findings		4.2 (0.6)	3.5 (0.7)	*p* = 0.02*
Carrying out procedures		3.8 (0.7)	3.6 (1.0)	*p* = 0.43
Suggesting differential diagnoses		3.5 (0.7)	3.7 (0.9)	*p* = 0.27
Suggesting further investigations		3.8 (0.4)	3.9 (0.7)	*p* = 0.56
Knowing which topics that I master		3.4 (0.6)	3.6 (0.9)	*p* = 0.34
Knowing which examinations that I master		3.8 (0.4)	3.7 (0.9)	*p* = 1.00
Knowing which procedures that I master		3.9 (0.5)	4.1 (0.6)	p = 0.34
**Confidence**^2^	**0.74**	**3.6 (0.6)**	**3.7 (0.7)**	**p = 0.84**
Not afraid of asking for help		4.2 (0.6)	4.4 (0.6)	*p* = 0.35
Not afraid of asking for feedback		3.7 (0.9)	3.6 (0.9)	p = 0.77
Confidence in performing tasks expected of a fifth-year medical student		3.2 (1.0)	3.2 (0.8)	*p* = 0.89
Confidence in having learned enough		3.3 (0.9)	3.4 (1.1)	p = 0.75
**Motivation**^2^	**0.30**	**3.6 (0.6)**	**3.5 (0.5)**	***p*** **= 0.23**
Motivation to meet/clerk patient		4.1 (0.8)	3.9 (0.7)	*p* = 0.49
Motivation to learn		3.8 (0.9)	3.6 (1.0)	p = 0.64
Regularly sought medical knowledge		3.1 (0.8)	2.8 (0.8)	p = 0.44

^1^*1* never, *2* rarely, *3* sometimes, *4* often, *5* always

^2^*1* strongly disagree, *2* disagree, *3* neutral, *4* agree, *5* strongly agree

*Note:* ^a^ denotes item that was reverse scored; * denotes items where difference was statistically significant at *p* < 0.05

## Discussion

In this study, formative mini-CEX assessments were compared to traditional ad-hoc feedback to examine student perceptions and effects on direct observation, feedback and learning outcomes. Students were positive towards the use of mini-CEX, and most found them helpful for their learning. We found no differences between the groups with regards to direct observation, feedback or learning outcome.

Implementation of formative mini-CEX assessments in an undergraduate clinical placement was feasible, and all participants met the pre-planned number of assessments. Assessments were completed in a mean of approximately 25 min, 20 min for observation and 5–6 min for feedback, which is in line with both the intention and the published research [8, 25]. The assessments covered a wide range of common clinical problems, and all participants met the pre-planned requirement of eight mini-CEX encounters. This is higher than completion rates reported in most other studies, with a recent systematic review finding mixed results but rates generally above 50% [5, 7, 13, 25]. This may be explained by several factors. Firstly, our study took place in an undergraduate setting, where doctors are already used to supporting students when seeing patients. Secondly, a small number of students per hospital and allowing all doctors to carry out assessments, thereby minimising workload per doctor. Thirdly, our participants typically spent seven weeks in the same rotation, which may have contributed to facilitating assessments. Short rotations have been found to make assessments and meaningful feedback more challenging, as trainees and supervisors do not get to know each other [26].

Despite the high completion rate, many participants commented that finding a time or suitable clinical setting was challenging, and assessors were often perceived to be busy. Feasibility issues relating to time constraints have been identified in numerous other studies [22, 26–28]. However, it is encouraging to see that only four participants reported that a doctor had refused to do an assessment. Previous recommendations for facilitating implementation of WBAs have emphasised the need for ensuring the necessary resources, including time and faculty development [26].

### Student perceptions

Most students were positive to the use of mini-CEX assessments and found them useful during their clinical placement. Participants recognised the importance of constructive feedback, and would have liked more feedback on areas of improvement. While most studies show that trainees value feedback and find assessments useful [4, 5, 29]; others found that trainees regard WBAs as a tick-box exercise or a waste of time [22, 30]. We did not find the latter in our study, possibly explained by the voluntary inclusion and emphasis on the assessments’ formative nature.

A number of participants did not feel confident that the mini-CEX assessments gave a true reflection of their capabilities. Similar results among junior doctors have been described previously [22]. This could reflect the students’ perception that feedback was limited, or a need to train assessors for accurate scoring. Previous research has shown that raters seldom use the full nine-point scale and leniency in scoring is common, which is also the case in our study [9].

### Effects on direct observation and feedback

Implementing formative mini-CEX assessments did not lead to reported increase of direct observation or feedback overall. Direct observation of clinical skills was reported as infrequent in both groups, and the majority were not satisfied with the amount of feedback they received. This may be explained by different expectations to or perceptions of what constitutes direct observation and feedback. The intervention group, having been introduced to the mini-CEX both through theory and practice, may have expected more of their feedback conversations in terms of both quantity and quality. In order to study the genuine difference, field studies are needed.

However, feedback on history taking was reported significantly more common in the intervention group. This is encouraging, as concerns have been raised over supervisors basing their assessments of trainees’ clinical skills on proxy information, such as inferring history takings skills based on the case presentation [31, 32]. Some participants highlighted the mini-CEX’s value in terms of ‘forcing’ observation and feedback, and this may be especially relevant for more time-consuming skills such as history taking.

Both groups indicated that junior doctors most frequently provided supervision and feedback, and some participants felt that feedback from more experienced doctors would be more useful. We know from previous research that credibility is an important determinant of how impactful feedback is [33, 34]. This includes trainees’ perceptions of supervisor characteristics such as experience [34]. However, this must be weighed against feasibility aspects. If direct observation and feedback can only be given by experienced doctors, workload on the few increases, and less experienced doctors are deprived of situations in which they can develop their skills as supervisors. This should also be supported by robust faculty development to improve their skills as educators.

### Educational impact

Educational impact can be classified according to Kirkpatrick’s framework, later adapted for medical education research by Barr and colleagues [35, 36]. In this study, we have presented both self-reported outcome measures (Kirkpatrick level 1) and impact on performance (Kirkpatrick level 2b). We found that for self-reported improvement in performing key tasks, such as history taking and clinical examination, there was no statistically significant difference between the groups overall. Interestingly though, the intervention group perceived their ability to identify normal and abnormal findings significantly higher than the control group. This may indicate that students use mini-CEX assessments as learning situations in which their clinical findings can be verified by a more experienced doctor. In this case, there is a recognised knowledge gap from the student’s point of view, and feedback given is both specific and actionable, and therefore more likely to be effective [37].

Performance on the OSCE and written test found slightly higher scores in the intervention group, though not statistically significant. This contrasts two previous studies that have shown positive effects on trainee performance, although none of these were randomised controlled studies [16, 17].

The inconsistent findings may be explained by several factors. Firstly, all studies have used general outcome measures, which may have left a large proportion of the effect invisible [25]. Secondly, it is logical to think that educational impact of the mini-CEX depends heavily on the quality of the feedback conversation following the assessment. Although we have little data with regards to the content in these conversations, we found that positive feedback was provided on over 80% of forms and suggestions for improvement in 70% of forms. The quality of feedback provided on WBA forms was the topic of a study by Vivekananda-Schmidt and colleagues, who found that only around 40% of forms contained free-text comments and goal-oriented feedback to support trainee development was uncommon [38]. Further research into the efficacy of formative mini-CEXs should also consider the quality of feedback conversations and its impact on learning.

### Strengths and weaknesses

There are several limitations to our study. The study is small and the effect size of approximately one standard deviation may be too large to be realistically expected of the intervention. Regrettably, we were not able to include the number of participants needed to achieve adequate power to evaluate the written test, as we did not have resources available to include additional hospitals in the study. The results from the written test are further limited by low reliability, most probably as a consequence of few items. Another limitation related to the analyses is that the increase in error across multiple comparisons was not controlled, but we consider the research preliminary and encourage replication of its findings. Additionally, generalisability may be limited by the study being a single-institution study. However, we believe that including both general medicine and surgery, as well as multiple hospitals, strengthen the generalisability of our findings. This is, to our knowledge, the first randomised controlled study of the effects of mini-CEX on direct observation, feedback and educational impact. The study included both self-reported and objective data on performance. Performance data was controlled for baseline competence in the form of scores from previous examinations, and scoring was blinded as to what group the participants belonged to.

## Conclusions

There is still considerable potential in assessing medical students during clinical placements and in routine practice, but the educational impact of formative assessments remains mostly unknown. We found that the mini-CEX is feasible and students are generally positive towards their use. However, we found no measurable effects with regards to overall feedback, or performance on summative tests. This study contributes to the ongoing discussion with a robust study design, and may serve as a basis for future research.

## Supplementary Information

**Additional file 1.** Characteristics of mini-CEX encounters.

## Data Availability

The datasets used and analysed in this study are available from the corresponding author on reasonable request.

## Abbreviations

WBA: Workplace-based assessment
mini-CEX: Mini-Clinical Evaluation Exercise
OSCE: Objective Structured Clinical Examination
MCQ: Multiple Choice Questions
ANOVA: One-way analysis of variance
ANCOVA: One-way analysis of covariance
STC: Systematic Text Condensation
