# Supplementary material for: Examining the educational impact of the mini-CEX: a randomised controlled study
Source: BMC Med Educ. 2021 Apr 21;21:228. doi: 10.1186/s12909-021-02670-3 (PMC8061047; doi:10.1186/s12909-021-02670-3)
Supplement: Supplementary file 1 — Additional file 1. Characteristics of mini-CEX encounters. [file 12909_2021_2670_MOESM1_ESM.docx]

**Additional file 1** Characteristics of mini-CEX encounters

|  |  |
| --- | --- |
| **Specialty**  General medicine, *N (%)*  General surgery and orthopaedics, *N (%)*  Anaesthesiology, *N (%)*  **Encounter type**  History taking and/or clinical examination, *N (%)*  Clinical case presentations, *N (%)*  Procedures, *N (%)*  Patient discharge conversations, *N (%)*  **Patient characteristics**  Age in years, *mean*^1^  Age in years, *range*^1^  Gender, *% female*^2^  **Most common patient presentations**^3^  *Abdominal pain, chest pain, shortness of breath,*  *fever, trauma*  **Most common patient diagnoses**^3^  *Appendicitis, atrial fibrillation, urinary tract*  *infection, pneumonia, epilepsy, gall stones,*  *chronic obstructive pulmonary disease,*  *fractures* | 87 (54.4)  69 (43.1)  4 (2.5)  130 (81.3)  15 (9.4)  12 (7.5)  3 (1.9)  62.3  0-90  50 |
| ^1^Information missing in 6% of encounters  ^2^Information missing in 1% of encounters  ^3^Information missing in 6% of encounters |  |
